# On Testing Dependence between Time to Failure and Cause of Failure when Causes of Failure Are Missing

**DOI:** 10.1371/journal.pone.0001255

**Published:** 2007-12-05

**Authors:** Isha Dewan, Sangita Kulathinal

**Affiliations:** 1 Indian Statistical Institute, New Delhi, India; 2 Indic Society for Education and Development, Nashik, India; University of East Piedmont, Italy

## Abstract

The hypothesis of independence between the failure time and the cause of failure is studied by using the conditional probabilities of failure due to a specific cause given that there is no failure up to certain fixed time. In practice, there are situations when the failure times are available for all units but the causes of failures might be missing for some units. We propose tests based on U-statistics to test for independence of the failure time and the cause of failure in the competing risks model when all the causes of failure cannot be observed. The asymptotic distribution is normal in each case. Simulation studies look at power comparisons for the proposed tests for two families of distributions. The one-sided and the two-sided tests based on Kendall type statistic perform exceedingly well in detecting departures from independence.

## Introduction

We consider a competing risks set-up where a unit is subject to two disjoint risks of failure and each unit ultimately fails due to either of the two risks. We do not allow simultaneous failures due to both the risks. The observations are made on the time to failure T and an identifier of the risk *δ* = *j* if the failure is due to the risk j, j = 0, 1. Let the joint distribution of (*T*,*δ*) be specified by the subsurvival functions *S_j_*(*t*) = *P*(*T*≥*t*, *δ* = *j*), or the subdistribution function given by *F_j_*(*t*) = *P*(*T*<*t*, *δ* = *j*), *j* = 0,1. The survival function and the distribution function of T are, respectively, given by
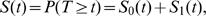


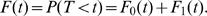
We assume that the subsurvival functions are continuous. Note that the distribution of the failure time and the cause of failure is specified using the observable variables (*T*,*δ*).

Let the conditional probability of failure due to the first risk given that there is no failure up to time *t* be given as
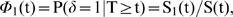
whenever *S*(*t*)>0. These probabilities were introduced while studying failure and preventive maintenance in a censoring setting where the interest is in the distribution of the failure time which would have been observed in the absence of preventive maintenance [1]. Another conditional probability of interest is
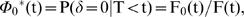
whenever *F*(*t*)>0.

Under independence *S_j_*(*t*) = *S*(*t*)*P*(*δ* = *j*) and hence, *T* and *δ* can be studied separately. Thus, the hypothesis of equality of subsurvival functions reduces to testing whether *P*(*δ* = 1) = *P*(*δ* = 0) = 1/2, a Bernoulli trial with success probability half. Hence a two-dimensional problem reduces to one-dimensional problem. The dependence between the failure time *T* and the cause of failure *δ* in terms of the above two conditional probability functions was studied in [2]. Below we give formal proofs of two results, which were stated in [2] on the independence and *positive quadrant dependent* (PQD) structure of (*T*,*δ*) in terms of these conditional probabilities.

**Lemma 1:** *T* and *δ* are independent if and only if Φ_1_(*t*) = Φ_1_(0) = *φ*, for all *t* where *φ* = *P*(*δ* = 1).

**Proof:** When *T* and *δ* are independent their joint distribution is written as the product of the marginal distributions. Hence,
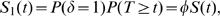


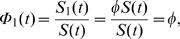
where *φ* = *P*(*δ* = 1). Similarly when Φ_1_(*t*) does not depend on *t* then Φ_1_(*t*) = Φ_1_(0) and Φ_1_(0) = *S*_1_(0)/*S*(0) = *P*(*δ* = 1) = *φ*.

This in turn implies that *S*_1_(*t*) = *φS*(*t*) which is the product of the marginal distributions of *δ* and *t*. Also, *S*_0_(*t*) = *S*(*t*)−*S*_1_(*t*) = (1−*φ*)*S*(*t*). Hence the result.

The independence of *T* and *δ* is also equivalent to Φ^*^_0_(*t*) = Φ^*^_0_(0), for all *t*. A simple and easily checked dependence structure is *positive quadrant dependent* (PQD) indicating positive association between two random variables.

**Definition 1:** Random variables X and Y are Positive Quadrant Dependent (PQD) if the following inequality holds:
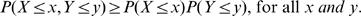
In our case, because *δ* takes only two values 0 and 1, *T* and *δ* are PQD if the following inequality holds:
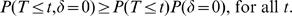
This is because *P*(*T*≤*t*,*δ*≤0) = *P*(*T*≤*t*,*δ* = 0), *P*(*T*≤*t*,*δ*≤1) = *P*(*T*≤*t*), and *P*(*δ*≤1) = 1. Hence the required inequality always holds for *δ* = 1.

**Lemma 2:** *T* and *δ* are PQD if and only if Φ_1_(*t*)≥Φ_1_(0) = *φ*, for all *t*.

**Proof:** When *T* and *δ* are PQD then
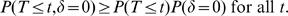
Note that
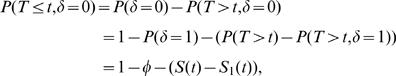
and *P*(*T*≤*t*)*P*(*δ* = 0) = (1−*S*(*t*))(1−*φ*). Substituting these identities in the above inequality, we get
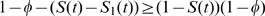


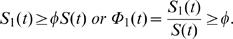
Hence the result.

Note that *T* and *δ* are *positive quadrant dependent* (PQD) is also equivalent to Φ^*^_0_(*t*)≥Φ^*^_0_(0), for all *t*>0. Various hypothesis testing problems of checking independence of *T* and *δ* against various alternatives specifying dependence structures are considered and U-statistics are derived when the complete data on all *n* units are available [2]. However, in many practical situations the experimenter may have information on failure times for all the individuals but on the causes of failures only for some.

In mortality follow-up study, the causes of death are obtained from the death certificates. The problem of causes of death missing on death certificates is well-known. This may occur due to various reasons like doctor's strike, autopsy not performed and hence, no knowledge of Definite underlying cause of death, and no legal requirement of mentioning an underlying cause of death on the death certificate. The present work is motivated by a follow-up study on mortality where the underlying causes of death were missing for nearly 20% patients but the times of death were known for all. Similar situation arises in engineering fields where series systems are tested for failure due to various components, possibly under accelerated life testing. In this case, a thorough autopsy of failed system is required to identify the failed component(s) which leads to the system failure. Such information may not be available for all failed systems due to financial or logistic reasons. In motorcycle fatalities study, 40% of the death certificates had either partial or no information on underlying causes of death [3].

An example from animal bioanalysis where all causes were not available was considered [4]. Likelihood based estimation in case of missing causes of failure have been studied [5], [6], [7], [8], [9]. A modified log rank test for competing risks with missing failure type was also considered [10]. The maximum likelihood estimators and minimum variance unbiased estimators of the parameters of exponential distribution for the missing case were obtained [11] and their approximate and asymptotic properties were discussed and confidence intervals were derived [12].

In this paper, we consider the problem of testing
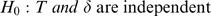
against various alternative hypotheses characterising the dependence structure of *T* and *δ*, which are:
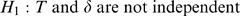


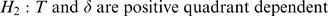
when causes are missing for some units. Let (*T_i_*,*δ_i_*), *i* = 1,…,*n* be the competing-risks data available on *n* individuals. Here, we consider a situation when *δ_i_* may not be observed always *i.e.,* it may be missing for some units. Let *O_i_* be an indicator variable which takes value one if *δ_i_* is observed and zero if *δ_i_* is missing. Let *p* = *pr*(*O_i_* = 1) be the probability that *δ* is observed. We assume that *δ_i_* are missing completely at random and hence *O_i_* is independent of (*T_i_*,*δ_i_*). The fact that the cause of failure will be observed or not will have no bearing on the actual cause. Similar assumptions are made in [9], [13].

We extend some of the tests based on U-statistics proposed in [2] to the case when *δ*′*s* are not observed for all the units. We carry out simulation studies for comparing the power of the tests for two families of distributions. We also apply the proposed tests to the data on failure of switches given in [14] by artificially creating missing causes of failure. The proposed tests perform satisfactorily and the use of the data on the failure times even when corresponding causes are missing is recommended.

## Results

We apply the proposed tests *U_km_*, *U_PQDm_* and *U_km_*_1_, the one-sided test based on *U_km_* to simulated data from two parametric families of distributions and evaluate empirical powers. We also apply the tests to a real data. The computations were done using SAS [15] and the source codes and a brief guide on how to use the SAS codes are provided in the supplementary material (Text S1, Text S2, Text S3 and Text S4).

**Example 1:** Parametric family of distributions [2]

Consider the parametric family of distributions proposed in [2]

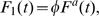


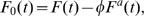
where 1≤*a*≤2, 0≤*φ*≤0.5 and *F*(*t*) is a proper distribution function. Note that *P*(*δ* = 1) = *φ* and
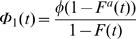
which is an increasing function of *t*. For *a* = 1, Φ_1_(*t*) = *φ*, that is, *T* and *δ* are independent and for 1<*a*≤2, Φ_1_(*t*)>*φ*, that is *T* and *δ* are PQD , and hence *H*_2_ holds. Let *F*(*t*) = 1−exp{−*λt*} be the overall distribution function. We simulated random samples by varying *n*, *p*, and (*λ,a,φ*) from the above distribution. Empirical level of significance and power were calculated by using 1000 replications for each combination of *n*, and (*λ*,*a*,*φ*). Table 1 gives the empirical level of significance and Table 2 gives the empirical power of the three tests based on *U_km_*, *U_km_*_1_ and U_PQDm_.

**Table 1:** Empirical level of significance of the three U-tests for a family of distributions of Dewan *et al.* (2004) (*λ*, *a*) = (1,1)

*φ*	*p*	*n* = 25			*n* = 50			*n* = 100		
		*U_km_*	*U_km1_*	*U_PQDm_*	*U_km_*	*U_km1_*	*U_PQDm_*	*U_km_*	*U_km1_*	*U_PQDm_*
0.5	1	0.057	0.054	0	0.061	0.049	0.001	0.052	0.047	0.002
0.5	0.9	0.055	0.048	0.002	0.062	0.052	0.001	0.047	0.053	0.004
0.5	0.8	0.054	0.047	0.007	0.049	0.061	0.008	0.05	0.049	0.004
0.2	1	0.069	0.061	0.001	0.052	0.049	0.001	0.051	0.06	0.01
0.2	0.9	0.078	0.059	0.001	0.061	0.052	0.002	0.052	0.059	0.005
0.2	0.8	0.092	0.06	0.005	0.057	0.054	0.004	0.047	0.064	0.002

**Table 2:** Empirical power of the three U-tests for a family of distributions of Dewan *et al.* (2004) *λ* = 1 and *φ* = 0.5

*φ*	*p*	*n* = 25			*n* = 50			*n* = 100		
		*U_km_*	*U_km1_*	*U_PQDm_*	*U_km_*	*U_km1_*	*U_PQDm_*	*U_km_*	*U_km1_*	*U_PQDm_*
1.5	1	0.421	0.546	0.049	0.690	0.812	0.201	0.942	0.974	0.575
1.5	0.9	0.394	0.505	0.066	0.652	0.767	0.201	0.916	0.955	0.504
1.5	0.8	0.365	0.489	0.079	0.609	0.711	0.208	0.894	0.938	0.453
1.8	1	0.765	0.863	0.183	0.967	0.988	0.646	0.999	0.999	0.974
1.8	0.9	0.713	0.802	0.181	0.947	0.975	0.545	0.998	0.999	0.917
1.8	0.8	0.653	0.768	0.191	0.921	0.965	0.485	0.997	0.999	0.836

From the two tables it is clear that modified test statistic *U_km_* attains its level when roughly half of the failures are likely to be due to the first cause. The conclusions are valid even when 20% of the failure causes are not available. The power increases with increase in values of *a* and also with increase in sample size. The test has very good power even when *a* = 1.5. One should keep in mind the fact that the alternative of no independence is extremely general.

However, the test based on one-sided version of the Kendall's *τ*, *U_km_*_1_ performs much better than the test based on *U_PQDm_* for testing *H*_0_ against *H*_2_. It was observed in [2] that the test *U_PQDm_*, when *p* = 1 is extremely conservative and also inefficient. The entries for this test in the two tables confirm this observation. But given the fact that the level of significance attained is very low, it is able to detect alternatives reasonably well.

**Example 2:** Random sign censoring model [1]

A random sign censoring (RSC), also known as an age-dependent censoring, is a model in which the lifetime of a unit (*X*) is censored by *Z* = *X*−*Wη*, where *W*, 0<*W*<*X* is the time at which a warning is emitted by the unit before its failure, and *η* is a random variable taking values {−1,1} and is independent of *X*. Hence *η* = 1 would lead to the censoring of the life time at *X*−*W* giving *T* = *Z* and *δ* = 0 and η = −1 will lead to the observation of complete lifetime *X*, giving *T* = *X* and *δ* = 1. Assume that *X* has exponential distribution with parameter *λ*. In this case, *P*(*Z*≥*t*, *Z*<*X*) = *P*(*X*−*W*≥*t*, *η* = 1) and *P*(*X*≥*t*, *X*<*Z*) = *P*(*X*≥*t*)*P*(*η* = −1). This gives Φ_1_(*t*) = *P*(*T*≥*t*, *δ* = 1)/*P*(*T*≥*t*) = *P*(*X*≥*t*, *η* = −1)/*P*(*X*≥*t*, *X*−*Wη*≥*t*).

Here *P*(*η* = −1) = 1−*P*(*η* = 1) = *P*(*δ* = 1) = *φ*. When *X* is exponentially distributed with parameter *λ* and *W* = *aX*, 0<*a*<1,
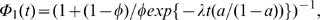
leading to the increasing (and hence PQD) nature of Φ_1_(*t*) in *t*. As *a* goes to zero Φ_1_(*t*) goes to *φ*, that is *T*,*δ* are independent. Hence, to evaluate the empirical level of significance we choose *a* very close to zero.

The value *a* close to zero corresponds to independence of *T* and *δ* and *a*>0 gives Φ_1_(*t*) as an increasing function of *t* implying *T* and *δ* are PQD. For simulation purposes, we consider two values of *a* = 0.00001, and 0.5. Test based on *U_km_* almost attains its level even for sample sizes as small as *n* = 25 as can be seen from Table 3. This test has good power for *n = *100. The test based on *U_km_*_1_ has a slightly higher level as well as higher power. But the test based on *U_PQDm_* is a very conservative test. It has low empirical power even for *n* = 100. One-sided test based on *U_km_*_1_ is definitely a better choice for detecting PQD alternatives.

**Table 3:** Empirical power of the three U-tests for random sign censoring model (*λ* = 1)

(*a*,*φ*)	*p*	*n* = 25			*n* = 50			*n* = 100		
		*U_km_*	*U_km_*_1_	*U_PQDm_*	*U_km_*	*U_km_*_1_	*U_PQDm_*	*U_km_*	*U_km_*_1_	*U_PQDm_*
(10^−5^_, _0.5)	1	0.057	0.067	0.002	0.061	0.063	0	0.052	0.065	0
(10^−5^_, _0.5)	0.9	0.055	0.065	0.001	0.062	0.059	0.001	0.047	0.053	0
(10^−5^_, _0.5)	0.8	0.054	0.059	0.005	0.049	0.059	0.008	0.05	0.048	0.006
(10^−5^_, _0.7)	1	0.056	0.057	0	0.067	0.063	0	0.056	0.057	0
(10^−5^_, _0.7)	0.9	0.063	0.069	0.005	0.059	0.064	0.003	0.055	0.06	0.003
(10^−5^_, _0.7)	0.8	0.062	0.067	0.009	0.057	0.063	0.006	0.052	0.056	0.000
(0.5, 0.5)	1	0.352	0.474	0.029	0.577	0.702	0.11	0.85	0.923	0.381
(0.5, 0.5)	0.9	0.321	0.442	0.05	0.513	0.647	0.135	0.793	0.884	0.358
(0.5, 0.5)	0.8	0.286	0.41	0.066	0.473	0.609	0.121	0.751	0.848	0.306
(0.5, 0.7)	1	0.32	0.423	0.029	0.489	0.61	0.091	0.759	0.847	0.303
(0.5, 0.7)	0.9	0.266	0.378	0.045	0.449	0.564	0.105	0.705	0.811	0.249
(0.5, 0.7)	0.8	0.229	0.348	0.056	0.396	0.516	0.112	0.655	0.755	0.233

**Example 3:** Nair's data revisited [14]

Here we consider the data on the failure of 37 switches due to one of the two possible causes of failures published in [14]. These data were analysed in [2] and it was shown that the failure time (*T*) and the cause of failure (*δ*)of switches were not independent. Also, the conditional probability of failure due to cause A, Φ_1_(*t*) was shown to be larger than *φ* and hence *T* and *δ* were PQD.

We calculate three test statistics for the entire data on 37 switches as earlier. We also artificially create missing data on the cause of failure for varying values of *p* and repeating it for 1000 times to evaluate the empirical powers of the test statistics.

The hypothesis of independence of *T* and *δ*, *H*_0_ is rejected against *H*_1_ at *α* = 5% level of significance using *U_km_* (the value of the test statistic is 2.70 which is larger than 1.96) and the one-sided test, *U_km_*_1_ (the value of the test statistic is 2.70 which is larger than 1.64) rejects the hypothesis *H*_0_ against *H*2 at *α* = 5% level of significance. However, the test based on PQD, *U_PQDm_* (the value of the test statistic is 1.35 which is smaller than 1.64) does not reject the hypothesis *H*_0_ against *H*_2_ at *α* = 5% level of significance. Table 4 shows the empirical powers of the tests for various values of *p*.

**Table 4:** Empirical power of the three U-tests for Nair's data (1993)

*p*	*U_km_*	*U_km1_*	*U_PQDm_*
0.9	0.962	0.993	0.127
0.8	0.841	0.945	0.167
0.7	0.705	0.865	0.18
0.6	0.578	0.758	0.187
0.5	0.454	0.638	0.172

As seen earlier with the simulated data, the test *U_km_*_1_ performs well even when 60% of the causes are missing. The power of *U_PQDm_* test is unsatisfactory.

## Discussion

Testing independence between the failure time *T* and the cause of failure *δ* is often important because of reduction in dimensionality and possibility of studying *T* and *δ* separately. The available tests use only completely observed data on *T* and *δ*. One cannot avoid missing data situation in practice and hence, the issue of the effect missing observations on the existing tests needs to be addressed.

From the simulation studies it is clear that the two-sided test, *U_km_* is performing well for both the families of distributions for sample sizes as small as 25 and when 20% of the causes of failure are not known. These observations can be made from Table 1, Table 2 and Table 3 with attained level of significance and high empirical power. The empirical powers for all the three tests are higher in the case of the parametric family of distributions of Example 1 compared to RSC model of Example 2 for all sample sizes. The performance of the one-sided test, *U_km_*_1_ based on Kedall's *τ* is clearly superior to the *U_PQDm_* as demonstrated by Table 1, Table 2 and Table 3. Even when all causes are known it observed that the test based on Kendall's *τ* is four times more efficient than the test based on *U_PQD_* [2]. Even in the case of missing causes, we recommend the use of *U_km_*_1_ for testing independence against PQD. One obvious reason is that Kendall's *τ* uses information on (*T*,*δ*) for each pair of observations. Similar observations are made on the basis of real data analysis of Example 3 (Table 4).

The failure times with missing information on causes of failures also provide useful information regarding departures from independence of *T* and *δ*, and hence, omitting such observations from the analysis may result in loss of efficiency. For this reason, the analysis may not be based on only the complete data on both time and causes of failures (with reduced sample size, which is random). This article is the first attempt of its kind to carry out the tests for independence under the assumption of missing completely at random. How the tests perform under the assumption of missing at random or even informative missingness remains an open research problem.

## Materials and Methods

### General dependence between *T* and *δ*

First we consider the problem of testing H_0_ : Φ_1_(*t*) = *φ*, for all *t* against H_1_ : Φ_1_(*t*) is not a constant, where Φ_1_(t) = P(δ = 1|T≥t) = S_1_(t)/S(t), and *φ* = Φ_1_(0) = *P*(*δ* = 1). Recall that a pair (*T_i_*,*δ_i_*) and (*T_j_*,*δ_j_*) is a concordant pair if *T_i_*>*T_j_*, *δ_i_* = 1, *δ_j_* = 0 or *T_i_*<*T_j_*, *δ_i_* = 0, *δ_j_* = 1 and is a discordant pair if *T_i_*>*T_j_*, *δ_i_* = 0, *δ_j_* = 1 or *T_i_*<*T_j_*, *δ_i_* = 1, *δ_j_* = 0. The *U*-statistic based on the idea of concordance and discordance pairs or Kendall's *τ* is
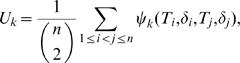
where
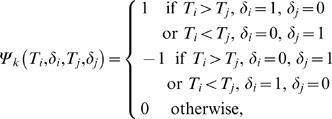
where the subscript *k* indicates that the *U*-statistic is defined using the idea of Kendall's *τ* [2]. If *δ* is missing for some units then *ψ_k_*(*T_i_*, *δ_i_*, *T_j_*, *δ_j_*) cannot be defined for all pairs. In Table 5 *m* indicates that *δ* is not observable and ? indicates the cases when *ψ_k_* is not defined.

**Table 5:** Values taken by the kernel *ψ_k_* for various combinations of the pairs (*T_i_*,*δ_i_*) and (*T_j_*,*δ_j_*)

*(δ_i_*,*δ_j_*)	(1,1)	(1,0)	(1,m)	(0,1)	(0,0)	(0,m)	(m,1)	(m,0)	(m,m)
*T_i_*>*T_j_*	0	1	?	−1	0	?	?	?	?
*T_i_*≤*T_j_*	0	−1	?	1	0	?	?	?	?

Note that when *T_i_*>*T_j_* and *δ_i_* = 1, but *δ_j_* is missing, *ψ_k_*(*T_i_*, *δ_i_*, *T_j_*, *δ_j_*) will take value 1 if *δ_j_* = 0 and value 0 if *δ_j_* = 1. Hence, in order to retrieve the best possible information we assign weight (1+0)/2 = 1/2 in this case. Similarly, when *T_i_*>*T_j_* and *δ_i_* = 0, but *δ_j_* is missing, *ψ_k_*(*T_i_*, *δ_i_*, *T_j_*, *δ_j_*) will take value −1 if *δ_j_* = 1 and value 0 if *δ_j_* = 0. Hence, we assign value −1/2 to the kernel in this case. Now, we redefine the kernel when some observations on *δ* are missing as follows
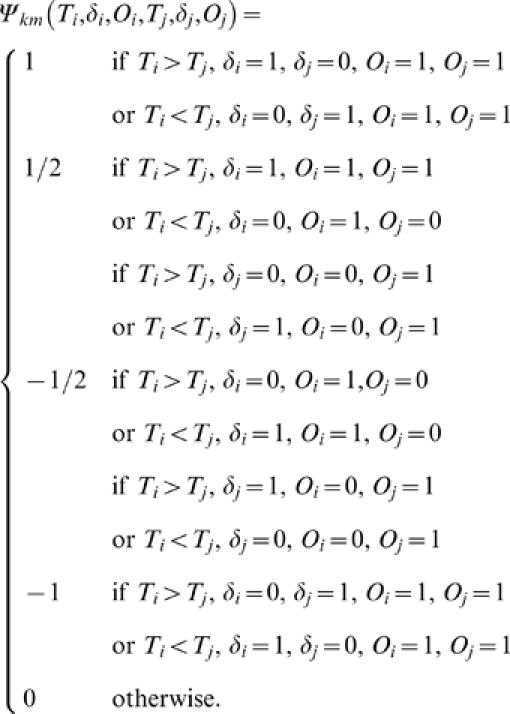
Here the subscript *m* indicates missing data situation. Define *U_km_* as the corresponding *U*-statistic
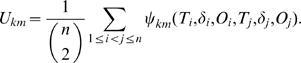
Then the expectations of *U_km_* is given by
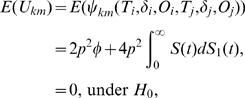
and the asymptotic variance of 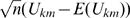 under *H*_0_, denoted as *Var*(*U_km_*) is
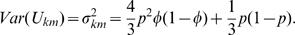
Note that when *p* = 1, the variance simplifies to (4/3)*φ*(1−*φ*), which is given in [2]. Also, *E*(*U_km_*)≠0 under *H*_1_. From the central limit theorem of *U*-statistics [16] (see Text S5), it follows that *U_km_* has asymptotic normal distribution for large n.

**Theorem 1:** Under *H*_0_, 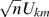 converges in distribution to *N*(0, σ^2^ *_km_*) as *n*→∞, where *σ* ^2^ *_km_* = (4/3)*p* ^2^ *φ*(1−*φ*)+(1/3)*p*(1−*p*).

We refer to the supplementary material (Text S6) for the explicit derivation of *E*(*U_km_*), *Var*(*U_km_*) and the proof of Theorem 1.

In practice, *p* and *φ* are generally unknown and can be replaced by their consistent estimators, 
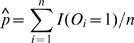
 and 
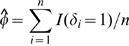
 respectively. A test procedure for testing *H*_0_ against *H*_1_ is then: reject *H*_0_ at 100*α*% level of significance if 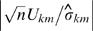 is larger than *z*_1−*α*_, cut-off point of standard normal distribution, where σ̂ ^2^ *_km_* is a consistent estimator of *σ* ^2^ *_km_* got by replacing *p* and *φ* by *p̂* and *φ̂*

For computational purposes, it is necessary to express *U_km_* as a function of ranks. Let 
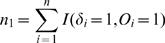
, 
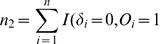
, and 
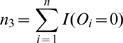
 represent numbers of observations in three groups-causes are observed to be 1, causes are observed to be 0 and causes are not observed, respectively. Let the corresponding ordered times in each group be represented by *X*_(1)_, *X*_(2)_,…, 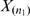, *Y*_(1)_, *Y*_(2)_,…, 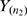, and *Z*_(1)_, *Z*_(2)_,…, 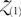, respectively. Let *R_i_* denote the combined rank of *X*_(*i*)_ in the ordered arrangement of (*n*_1_+*n*_2)_ samples of type *X* and *Y*, *S_i_* denote the combined rank of *X*_(*i*)_ in the ordered arrangement of (*n*_1_+*n*_3_) samples of type *X* and *Z*, and *Q_i_* denote the combined rank of *Y*_(*i*)_ in the ordered arrangement of (*n*_2_+*n*_3_) samples of type *Y* and *Z*. Then, the number of observations for which *ψ_km_*(.)takes value
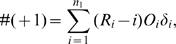


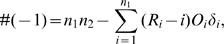


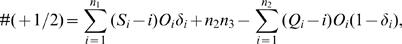


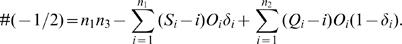
Thus, the expression *U_km_* in terms of ranks is
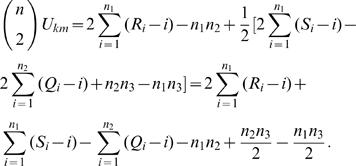
(1)


Consider testing *H*_0_ against *H*_2_. The *U*-statistic for testing *H*_0_ against *H*_2_ is
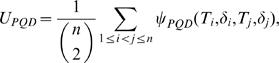
where
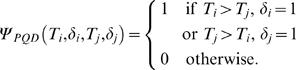
This test was proposed in [2]. If *δ* is missing for some units then *ψ_PQD_*(*T_i_*, *δ_i_*, *T_j_*, *δ_j_*) cannot be defined for all pairs. Table 6 shows the pairs for which the kernel is defined completely and also the cases where it is not defined.

**Table 6:** Values taken by the kernel *ψ_PQD_* for various combinations of the pairs (*T_i_*,*δ_i_*) and (*T_j_*,*δ_j_*)

(*δ_i_*,*δ_j_*)	(1,1)	(1,0)	(1,m)	(0,1)	(0,0)	(0,m)	(m,1)	(m,0)	(m,m)
*T_i_*≤*T_j_*	1	1	1	0	0	0	?	?	?
*T_i_*>*T_j_*	1	0	?	1	0	?	1	0	?

As in the earlier subsection, we define a modified kernel to take into account missing causes as follows.
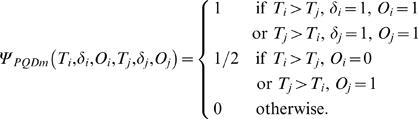
Let the corresponding *U*-statistic be *U_PQDm_*

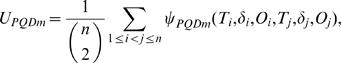
where the subscript *m* indicates missing data situation. The expectations of *U_PQDm_* are given by
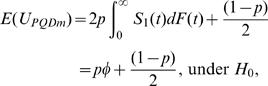
and 
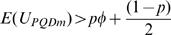
 under *H*_2_. The asymptotic variance of 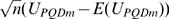 under *H*_0_, denoted as *Var*(*U_PQDm_*) is
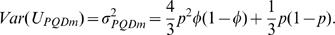
Note that when *p* = 1, the variance simplifies to (4/3)*φ*(1−*φ*), which is given in [2]. From the central limit theorem of U-statistics [16] (see Text S5), it follows that *U_PQDm_* has asymptotic normal distribution for large *n*.

**Theorem 2:** Under *H*_0_, 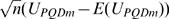 converges in distribution to *N*(0,*σ* ^2^ *_PQDm_*), where *σ* ^2^ *_PQDm_* = (4/3)*p* ^2^ *φ*(1−*φ*)+(1/3)*p*(1−*p*), as *n*→∞.

We refer to the supplementary material (Text S7) for the explicit derivation of E(U_PQDm_), *Var*(*U_PQDm_*) and the proof of Theorem 2.

We reject the null hypothesis for large values of 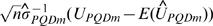 where *E*(*Û* *_PQDm_*) and *σ̂* *_PQDm_* are obtained by replacing *φ* and *p* by their empirical estimators. As mentioned earlier, *p* and *φ* can be replaced by their consistent estimators 
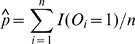
 and 
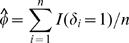
. Let *R_i_* ^*^ denote the rank of *T_i_* in the ordered observations (*T*_1_, *T*_2_, …, *T_n_*). Then, it is easy to see that
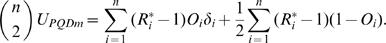
Note that a one-sided test based on *U_km_*, where *H*_0_ is rejected for large values of *U_km_* can also be used for testing *H*_0_ against *H*_2_ since *E*(*U_km_*)≥0 under *H*_2_. In fact, the one-sided test uses data on both the *T* and *δ* in each pairwise comparison while *U_PQDm_* uses only information on (*T*, *δ*) from one and *T* from the other in a pairwise comparison. We refer the one-sided test based on *U_km_* as *U_km_*_1_.

## Supporting Information

Text S1 SAS source code for Example 1
(0.06 MB DOC)

Text S2 SAS source code for Example 2
(0.06 MB DOC)

Text S3 SAS source code for Example 3
(0.03 MB DOC)

Text S4 A short guide on the use of SAS codes
(0.02 MB DOC)

Text S5 Central limit theorem for U-statistics
(0.07 MB DOC)

Text S6 Derivation of E(Ukm), Var(Ukm) and proof of Theorem 1
(0.14 MB DOC)

Text S7 Derivation of E(UPQDm), Var(UPQDm) and proof of Theorem 2
(0.07 MB DOC)
