# Supplementary material for: On Testing Dependence between Time to Failure and Cause of Failure when Causes of Failure Are Missing
Source: PLoS One. 2007 Dec 5;2(12):e1255. doi: 10.1371/journal.pone.0001255 (PMC2092381; doi:10.1371/journal.pone.0001255)
Supplement: Text S1 — SAS source code for Example 1 (0.06 MB DOC) [file pone.0001255.s001.doc]

**Text S1: SAS source code for Example 1**

The SAS source code for computing empirical level of significance and empirical power for the three tests when the data are simulated using the parametric distribution given in Example 1 is given here. The parametric distribution is

| 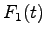 | = | 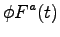 |
| --- | --- | --- |
| 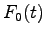 | = | 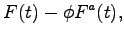 |
| 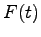 | = | 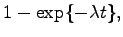 |
| 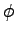 | = | 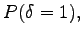 |
| 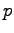 | = | 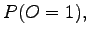 |
| 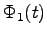 | = | 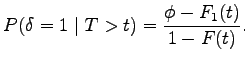 |

In the program below, (l1, w, a, p, nsample) represent the model parameters where $n$is the sample size. The argument rep in the program is the number of replications required. Choose $a$ in the interval $[1,2]$ and $a=1$ corresponds to the null hypothesis $H_{0}$of independence of $T$and $\delta.$

%MACRO example1(l1,w,a,p,nsample,rep);

%LET iter = 1;

%DO %UNTIL (&iter=&rep.);

%LET iter = %eval(&iter+1);

DATA gumbel(KEEP=t d o);

DO j = 1 TO &nsample.;

/* Generate random sample from exponential distribution */

t = (-1/&l1.)*log(1-RANUNI(8+5*&iter));

/* Generate d from the conditional distribution of d given t */

y = 1 - &w.*&a.*(1 - exp(-&l1.*t))**(&a. - 1);

IF RANUNI(9000 + 5*&iter) <= y THEN d = 0;

ELSE d = 1;

IF RANUNI(900+5*&iter) <= 1 - &p. THEN o = 0;

ELSE o = 1;

OUTPUT;

END;

RUN;

PROC SORT DATA=gumbel;BY t;RUN;

DATA gumbel;

SET gumbel;

BY t;

rank = _n_; /* Ranks of T's from the entire sample */

pqd1 = (rank - 1)*o*d;

pqd2 = (rank - 1)*(1 - o)*0.5;

pqd = (pqd1 + pqd2)*(2/(&nsample.*(&nsample. - 1)));

RUN;

/*-Calculation of Kendall's Tau U-satat-*/

PROC SORT DATA = gumbel(WHERE = (d = 1 AND o = 1)) OUT = x;BY t;RUN;

PROC SORT DATA = gumbel(WHERE = (d = 0 AND o = 1)) OUT = y;BY t;RUN;

PROC SORT DATA = gumbel(WHERE = (o = 1)) OUT = xy;BY t;RUN;

PROC SORT DATA = gumbel(WHERE = ((d = 1 AND o = 1) OR (o = 0))) OUT = xz;BY t;RUN;

PROC SORT DATA = gumbel(WHERE = ((d = 0 AND o = 1) OR (o = 0))) OUT = yz;BY t;RUN;

DATA x;

SET x;

rankx = _n_;

RUN;

DATA xy;

SET xy;

rankxy = _n_;

RUN;

DATA xz;

SET xz;

rankxz = _n_;

RUN;

DATA y;

SET y;

ranky = _n_;

RUN;

DATA yz;

SET yz;

rankyz = _n_;

RUN;

DATA termx;

MERGE x xy xz;

BY t;

term1 = 2*(rankxy - rankx) + (rankxz - rankx);

RUN;

DATA termy;

MERGE y yz;

BY t;

term2 = -1*(rankyz - ranky);

RUN;

PROC SORT DATA = gumbel;BY t;RUN;

PROC SORT DATA = termx;BY t;RUN;

PROC SORT DATA = termy;BY t;RUN;

DATA gumbel;

MERGE gumbel termx termy;

BY t;

n1 = o*d;

n2 = o*(1 - d);

n3 = 1 - o;

RUN;

PROC MEANS DATA = gumbel SUM NOPRINT;

OUTPUT out = ustat SUM =;

VAR PQD o d n1 n2 n3 term1 term2;

RUN;

DATA ustat(KEEP = lambda prob1 a probo n repeat upqd ukendall);

SET ustat;

/* variance of PQD and Kendall's U-stats */

var = (4/3)*(o/&nsample.)*(o/&nsample.)*(d/&nsample.)*(1 - d/&nsample.)

+ (1/3)*(o/&nsample.)*(1 - o/&nsample.);

/* expectation of PQD U-stat */

epqd = (o/&nsample.)*(d/&nsample.) + (1 - o/&nsample.)/2;

upqd = sqrt(&nsample./var)*(pqd - epqd);

ukendall = sqrt(&nsample./var)*(term1 + term2 - n1*n2 + n2*n3/2 - n1*n3/2)

*(2/(&nsample.*(&nsample. - 1)));

lambda = &l1.;

prob1 = &w.;

a = &a.;

probo = &p.;

n = &nsample.;

repeat = &rep.;

RUN;

/*-Dataset containing normalised test statistics of each iteration-*/

PROC datasets;

APPEND BASE = ustat_dep DATA = ustat FORCE;

RUN;

%END;

%MEND;

/*-Macro call here-*/

%example1(1,0.5,1,1,25,1001); /* One set of parameters */

title'U-statistics values';

PROC PRINT DATA = ustat_dep;RUN;

/*-Power calculations-*/

DATA power_u;

SET ustat_dep;

IF upqd > 1.64 THEN power_pqd = 1;

ELSE power_pqd=0;

IF ukendall > 1.96 OR ukendall < -1.96 THEN power_ken = 1;

ELSE power_ken = 0;

IF ukendall > 1.64 THEN power_ken1 = 1;

ELSE power_ken1 = 0;

RUN;

PROC SORT DATA = power_u;BY lambda prob1 a probo n repeat;RUN;

PROC MEANS DATA = power_u MEAN;

BY lambda prob1 a probo n repeat;

OUTPUT OUT = empower_ustat MEAN =;

VAR power_pqd power_ken power_ken1;

RUN;

title'Empirical powers of three U-tests';

PROC PRINT DATA = empower_ustat;RUN;
