# Supplementary material for: On Testing Dependence between Time to Failure and Cause of Failure when Causes of Failure Are Missing
Source: PLoS One. 2007 Dec 5;2(12):e1255. doi: 10.1371/journal.pone.0001255 (PMC2092381; doi:10.1371/journal.pone.0001255)
Supplement: Text S3 — SAS source code for Example 3 (0.03 MB DOC) [file pone.0001255.s003.doc]

**Text S3: SAS source code for Example 3**

The SAS source code for testing independence of $T$and $\delta$ for Nair's data using the three tests is given here. The missing data are artificially generated for various values of $p.$ The input to the arguments of the SAS macro is the name of the input SAS dataset and the size of the sample.

%MACRO ustat(indat,nsample);

/*-Creating missing data indicator-*/

%DO i=5 %TO 10 %BY 1;

%LET p = &i./10;

%DO rep = 1 %TO 1000;

DATA gumbel(KEEP = t d o);

SET &indat.;

IF RANUNI(900 + 5*&rep.) <= 1 - &p. THEN o = 0;

ELSE o = 1;

RUN;

PROC SORT DATA = gumbel;BY t;RUN;

DATA gumbel;

SET gumbel;

BY t;

rank = _n_; /* Ranks of T's from the entire sample */

pqd1 = (rank - 1)*o*d;

pqd2 = (rank - 1)*(1 - o)*0.5;

pqd = (pqd1 + pqd2)*(2/(&nsample.*(&nsample. - 1)));

RUN;

/*-Calculation of Kendall's Tau U-satat-*/

PROC SORT DATA = gumbel(WHERE = (d = 1 AND o = 1)) OUT = x;BY t;RUN;

PROC SORT DATA = gumbel(WHERE=(d = 0 AND o = 1)) OUT = y;BY t;RUN;

PROC SORT DATA = gumbel(WHERE = (o = 1)) OUT = xy;BY t;RUN;

PROC SORT DATA = gumbel(WHERE = ((d = 1 AND o = 1) OR (o = 0))) OUT = xz;BY t;RUN;

PROC SORT DATA=gumbel(WHERE = ((d = 0 AND o = 1) OR (o = 0))) OUT = yz;BY t;RUN;

DATA x;

SET x;

rankx = _n_;

RUN;

DATA xy;S

ET xy;

rankxy = _n_;

RUN;

DATA xz;

SET xz;

rankxz = _n_;

RUN;

DATA y;

SET y;

ranky = _n_;

RUN;

DATA yz;

SET yz;

rankyz = _n_;

RUN;

DATA termx;

MERGE x xy xz;

BY t;

term1 = 2*(rankxy - rankx) + (rankxz - rankx);

RUN;

DATA termy;

MERGE y yz;

BY t;

term2 = -1*(rankyz - ranky);

RUN;

PROC SORT DATA = gumbel;BY t;RUN;

PROC SORT DATA = termx;BY t;RUN;

PROC SORT DATA = termy;BY t;RUN;

DATA gumbel;

MERGE gumbel termx termy;

BY t;

n1 = o*d;

n2 = o*(1 - d);

n3 = 1 - o;

RUN;

PROC MEANS DATA = gumbel SUM NOPRINT;

OUTPUT out = ustat SUM =;

VAR PQD o d n1 n2 n3 term1 term2;

RUN;

DATA ustat(KEEP = probo repeat upqd ukendall);

SET ustat;

/* variance of PQD and Kendall's U-stats */

var = (4/3)*(o/&nsample.)*(o/&nsample.)*(d/&nsample.)*(1 - d/&nsample.)

+ (1/3)*(o/&nsample.)*(1 - o/&nsample.);

/* expectation of PQD U-stat */

epqd = (o/&nsample.)*(d/&nsample.) + (1 - o/&nsample.)/2;

upqd = sqrt(&nsample./var)*(pqd - epqd);

ukendall = sqrt(&nsample./var)*(term1 + term2 - n1*n2 + n2*n3/2 - n1*n3/2)

*(2/(&nsample.*(&nsample. - 1)));

probo = &p.;

repeat = &rep.;

RUN;

/*-Dataset containing normalised test statistics of each iteration-*/

PROC datasets;

APPEND BASE = ustat_dep DATA = ustat FORCE;

RUN;

PROC datasets LIBRARY = work;

DELETE ustat termx termy x xy xz y yz;

RUN;

%END;

%END;

%MEND;

/*-Reading real data (replace this step with the data to be analysed)-*/

DATA nair;

INPUT timea $ di @@;

CARDS;

1.151 0 1.577 0 2.015 0 2.199 0 2.547 1

1.17 0 1.584 0 2.076 0 2.227 1 2.548 1

1.248 0 1.677 1 2.109 1 2.25 0 2.738 0

1.331 0 1.695 1 2.116 0 2.254 1 2.794 1

1.381 0 1.71 1 2.119 0 2.261 0 2.91 1

1.499 1 1.955 0 2.135 1 2.349 0 3.015 1

1.508 0 1.965 1 2.197 1 2.369 1 3.017 1

1.534 0 2.012 0

;

RUN;

DATA all(DROP = timea di);

SET nair;

t = timea*1;

d = di*1;

RUN;

/ --- Macro call --- */

%ustat(all,37);

title'U-statistics values';

PROC PRINT DATA = ustat_dep;RUN;

DATA power_u;

SET ustat_dep;

IF upqd > 1.64 THEN power_pqd = 1;

ELSE power_pqd = 0;

IF ukendall > 1.96 OR ukendall < -1.96 THEN power_ken = 1;

ELSE power_ken = 0;

IF ukendall > 1.64 THEN power_ken1 = 1;

ELSE power_ken1 = 0;

RUN;

PROC SORT DATA = power_u;BY probo;RUN;

PROC MEANS DATA = power_u MEAN;

BY probo;

OUTPUT OUT = empower_ustat MEAN =;

VAR power_pqd power_ken power_ken1;

RUN;

title'Empirical powers for three U-statistics --- */

PROC PRINT DATA = empower_ustat;RUN;
