# Supplementary material for: On Testing Dependence between Time to Failure and Cause of Failure when Causes of Failure Are Missing
Source: PLoS One. 2007 Dec 5;2(12):e1255. doi: 10.1371/journal.pone.0001255 (PMC2092381; doi:10.1371/journal.pone.0001255)
Supplement: Text S4 — A short guide on the use of SAS codes (0.02 MB DOC) [file pone.0001255.s004.doc]

**Text S4: A short guide on the use of SAS codes**

Each SAS code has a macro which could be called for various combinations of the parameters. As an example, one macro call is shown in each program and the users can add several macro calls with different sets of parameters. The results from each macro call are stored under the same data set (ustat_dep). This data set gives the values of the three U-statistics which is further used in computation of the empirical power. The data set giving the empirical powers (empower_ustat) of the three tests are then printed in an output file. The codes can be executed in SAS version 8 or higher [1].

**References**

[1] SAS Institute Inc. SAS/STAT users guide, version 8, 2000.
