# Supplementary material for: On Testing Dependence between Time to Failure and Cause of Failure when Causes of Failure Are Missing
Source: PLoS One. 2007 Dec 5;2(12):e1255. doi: 10.1371/journal.pone.0001255 (PMC2092381; doi:10.1371/journal.pone.0001255)
Supplement: Text S5 — Central limit theorem for U-statistics (0.07 MB DOC) [file pone.0001255.s005.doc]

**Text S5: Central limit theorem for U-statistics**

The central limit theorem (CLT) for U-statistics is stated as follows [1].

Let $(X_{1},Y_{1}),\ldots,(X_{n},Y_{n})$denote a random sample from a bivariate population. Let $\gamma$ be an estimable parameter of degree $M$with symmetric kernel$\psi((x_{1},y_{1}),(x_{2},y_{2}),\ldots,(x_{M},y_{M}))$.Let the U-statistic be given by

| 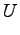 | = | 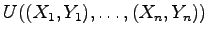 |
| --- | --- | --- |
|  | = | 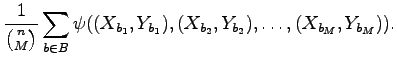 |

where $B$consists of all subsets of $M$integers chosen without replacement from $\{1,2,\ldots,n\}$and $M$ is the degree of the kernel. Let $E(U)$denote the expectation of *U.*

If$E(\psi^{2}((X_{1},Y_{1}),\ldots,(X_{M},Y_{M})))<\text{ }\infty$, then $\sqrt{n}(U-E(U))$has a limiting normal distribution (as$n\to\infty$) with mean zero and variance $M^{2}\eta_{1},$provided $\eta_{1}>0,$where $\eta_{1}$ is defined as

| 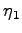 | = | 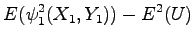 |
| --- | --- | --- |
| 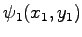 | = | 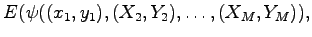 |

where $\psi_{1}(x_{1},y_{1})$ is the expectation of the kernel $\psi((X_{1},Y_{1}),\ldots,(X_{M},Y_{M}))$given the first pair of observations as $(x_{1},y_{1}).$This theorem also gives a simple way to derive the limiting variance of the *U*-statistic. We first derive the function similar to $\psi_{1}(x_{1},y_{1})$and then $\eta_{1}$ to obtain the limiting variances of $U_{km}$and $U_{PQDm}.$

**References**

[1] Puri ML and Sen PK (1971) Nonparametric methods in multivariate analysis: John Wiley,

New York-London-Sydney.
