# Supplementary material for: On Testing Dependence between Time to Failure and Cause of Failure when Causes of Failure Are Missing
Source: PLoS One. 2007 Dec 5;2(12):e1255. doi: 10.1371/journal.pone.0001255 (PMC2092381; doi:10.1371/journal.pone.0001255)
Supplement: Text S6 — Derivation of E(Ukm), Var(Ukm) and proof of Theorem 1 (0.14 MB DOC) [file pone.0001255.s006.doc]

**Text S6: Derivation of $E(U_{km}),$ $Var(U_{km})$ and proof of Theorem 1**

$E(U_{km})$is given as under.

$$E(U_{km})=E(\psi_{km}(T_{i},\delta_{i},O_{i},T_{j},\delta_{j},O_{j}))$$

$$=2P(T_{i}>T_{j}, \delta_{i}=1,\delta_{j}=0,O_{i}=1,O_{j}=1)$$

+

$$-P(T_{i}>T_{j}, \delta_{i}=0,O_{i}=1,O_{j}=0)-P(T_{i}<T_{j}, \delta_{i}=1,O_{i}=1,O_{j}=0)$$

$$-2P(T_{i}>T_{j}, \delta_{i}=0,\delta_{j}=1,O_{i}=1,O_{j}=1).$$

Note that

$$P(T_{i}>T_{j}, \delta_{i}=1,\delta_{j}=0,O_{i}=1,O_{j}=1)$$

$$=\text{ }P(O_{i}=1)P(O_{j}=1)P(T_{i}>T_{j}, \delta_{i}=1,\delta_{j}=0)$$

$$=\text{ }p^{2}\int_{0}^{\infty} \int_{0}^{t_{i}} dF_{0}(t_{j})dF_{1}(t_{i})$$

$$=\text{ }p^{2}\int_{0}^{\infty} F_{0}(t_{i})dF_{1}(t_{i})=p^{2}\int_{0}^{\infty} F_{0}(t)dF_{1}(t).$$

The first equality follows because the missingness is completely at random. All the other probabilities are computed similarly. The expression for $E(U_{km})$can be written as

$$E(U_{km})$$

$$=\text{ }2p^{2}\int_{0}^{\infty} F_{0}(t)dF_{1}(t)+p(1-p)\int_{0}^{\infty} F(t)dF_{1}(t)+p(1-p)\int_{0}^{\infty} S(t)dF_{0}(t)$$

$$-p(1-p)\int_{0}^{\infty} F(t)dF_{0}(t)-p(1-p)\int_{0}^{\infty} S(t)dF_{1}(t)-2p^{2}\int_{0}^{\infty} S_{0}(t)dF_{1}(t)$$

$$-p(1-p)\int_{0}^{\infty} (1-2S(t))dS_{1}(t)-p(1-p)\int_{0}^{\infty} (2S(t)-1)(-dS(t)+dS_{1}(t))$$

Under $H_{0},$ and hence, the integral in $E(U_{km})$ simplifies to This reduces $E(U_{km})$ to zero under $H_{0}.$ For carrying out the test procedure we need to work out the asymptotic variance of the *U*-statistic only under the null hypothesis of independence of $T$and $\delta.$Note that

$$E(\psi_{km}^{2}(T_{i},\delta_{i},O_{i},T_{j},\delta_{j},O_{j}))$$

$$=2P(T_{i}>T_{j}, \delta_{i}=1,\delta_{j}=0,O_{i}=1,O_{j}=1)$$

$$+\frac{1}{2}P(T_{i}>T_{j}, \delta_{i}=1,O_{i}=1,O_{j}=0)$$

$$+\frac{1}{2}P(T_{i}<T_{j}, \delta_{i}=0,O_{i}=1,O_{j}=0)$$

$$+\frac{1}{2}P(T_{i}>T_{j}, \delta_{i}=0,O_{i}=1,O_{j}=0)$$

$$+\frac{1}{2}P(T_{i}<T_{j}, \delta_{i}=1,O_{i}=1,O_{j}=0)$$

$$+2P(T_{i}>T_{j}, \delta_{i}=0,\delta_{j}=1,O_{i}=1,O_{j}=1),$$

and this is finite. Hence, the conditions of the CLT are satisfied and the limiting distribution of $\sqrt{n}(U_{km}-E(U_{km}))$ under $H_{0}$ is as stated in Theorem 1. We need to compute the asymptotic variance of $U_{km}$. Following the CLT, fix the $i$th triplet $(T_{i},\delta_{i},O_{i})$at $(t,\delta,O)$and under $H_{0}$, the conditional expectation of the kernel given the $i$th triplet is

where $I(A)$is an indicator function of event $A$ which takes value $1$ if $A$ holds true and is $0$otherwise. Now,

$$E(\psi_{km1}^{2}(T,\delta,O))$$

Solving the integrals, we get the limiting variance of $\sqrt{n}(U_{km}-E(U_{km})),$denoted as $Var(U_{km})$is
