# Supplementary material for: On Testing Dependence between Time to Failure and Cause of Failure when Causes of Failure Are Missing
Source: PLoS One. 2007 Dec 5;2(12):e1255. doi: 10.1371/journal.pone.0001255 (PMC2092381; doi:10.1371/journal.pone.0001255)
Supplement: Text S7 — Derivation of E(UPQDm), Var(UPQDm) and proof of Theorem 2 (0.07 MB DOC) [file pone.0001255.s007.doc]

**Text S7: Derivation of $\text{E}\left(\text{U}_{\text{PQDm}}\right)$, $\text{Var}\left(\text{U}_{\text{PQDm}}\right)$ and proof of Theorem 2**

We obtain $\text{E}\left(\text{U}_{\text{PQDm}}\right)$ similarly.

$$E(U_{PQDm})=E(\Psi_{PQDm}(T_{i},\delta_{i},O_{i},T_{j},\delta_{j},O_{j}))$$

$$=2pP(T_{i}>T_{j},\delta_{i}=1)+(1-p)P(T_{i}>T_{j})$$

$$=2p\int_{0}^{\infty} S_{1}(t)dF(t)+(1-p)/2$$

Note that

$$E(\Psi_{PQDm}^{2}(T_{i},\delta_{i},O_{i},T_{j},\delta_{j},O_{j}))=2pP(T_{i}>T_{j},\delta_{i}=1)+\frac{2}{4}(1-p)P(T_{i}>T_{j}),$$

and this is finite. Hence, the conditions of the CLT are satisfied and the limiting distribution of $\sqrt{n}(U_{PQDm}-E(U_{PQDm}))$under $\text{H}_{0}\text{ }$is as stated in Theorem 2. We need to compute the asymptotic variance of $\text{U}_{\text{PQDm}}$. Following the CLT, fix the$\text{ i }$th triplet $(T_{i},\delta_{i},O_{i})$ at $(t,\delta,O)$ and under $\text{H}_{0}\text{ }$, the conditional expectation of the kernel given the$\text{ i }$th triplet is

where $\text{I}\left(\text{A}\right)$ is an indicator function of event $A\text{ }$which takes value $1$ if$A$ holds true and is $0$otherwise. Now,

Solving the integrals, we get the limiting variance of $\sqrt{n}(U_{PQDm}-E(U_{PQDm}))$, denoted as $\text{Var}\left(\text{U}_{\text{PQDm}}\right)\text{ }$ is

.
